# Purinergic regulation of vascular endothelial growth factor signaling in angiogenesis

**DOI:** 10.1038/sj.bjc.6604998

**Published:** 2009-04-14

**Authors:** S M Rumjahn, N Yokdang, K A Baldwin, J Thai, I L O Buxton

**Affiliations:** 1 Department of Pharmacology, University of Nevada School of Medicine, Reno, NV 89557, USA

**Keywords:** breast cancer, angiogenesis, purinergic receptor, P2Y, VEGF, VEGFR2, phosphotyrosine

## Abstract

P2Y purine nucleotide receptors (P2YRs) promote endothelial cell tubulogenesis through breast cancer cell-secreted nucleoside diphosphate kinase (NDPK). We tested the hypothesis that activated P2Y_1_ receptors transactivate vascular endothelial growth factor receptor (VEGFR-2) in angiogenic signaling. P2Y_1_R stimulation (10 *μ*M 2-methyl-thio-ATP (2MS-ATP)) of angiogenesis is suppressed by the VEGFR-2 tyrosine kinase inhibitor, SU1498 (1 *μ*M). Phosphorylation of VEGFR-2 by 0.0262 or 2.62 nM VEGF was comparable with 0.01 or 10 *μ*M 2MS-ATP stimulation of the P2Y_1_R. 2MS-ATP, and VEGF stimulation increased tyrosine phosphorylation at tyr1175. 2MS-ATP (0.1–10 *μ*M) also stimulated EC tubulogenesis in a dose-dependent manner. The addition of sub-maximal VEGF (70 pM) in the presence of increasing concentrations of 2MS-ATP yielded additive effects at 2MS-ATP concentrations <3 *μ*M, whereas producing saturated and less than additive effects at ⩾3 *μ*M. We propose that the VEGF receptor can be activated in the absence of VEGF, and that the P2YR–VEGFR2 interaction and resulting signal transduction is a critical determinant of vascular homoeostasis and tumour-mediated angiogenesis.

The secretion of nucleoside diphosphate kinase (NDPK; EC 2.7.4.6) orthologues by intracellular parasites (Gounaris *et al*, 2001; Chopra *et al*, 2003), NDPK secretion by various carcinomas (Anzinger *et al*, 2001; Okabe-Kado and Kasukabe, 2003), and NDPK's extracellular role in blood flow regulation (Buxton *et al*, 2001) first lead us to propose a pathological role for secreted NDPK in cancer and tumour angiogenesis. We have recently provided evidence for a nucleotide-dependent regulation of angiogenesis by breast cancer-secreted NDPK (Rumjahn *et al*, 2007). We observed that extracellular NDPK by regeneration of extracellular nucleotides can use endothelial P2 (Y) nucleotide receptors to stimulate angiogenesis. Supporting our findings, the disruption of CD39 (ecto-apyrase EC 3.6.1.5) activity, the dominant vascular ecto-nucleotidase and its regulation of nucleotide signaling, has been observed to inhibit tumour angiogenesis and metastasis (Goepfert *et al*, 2001; Jackson *et al*, 2007). The regulation of extracellular ATP and ADP levels by ecto-apyrase is also known to play important roles in cardiovascular physiology and pathophysiology by activation of purinergic type-2 (P2) nucleotide receptors (Erlinge and Burnstock, 2008).

P2 nucleotide receptors activated by ATP include both ligand-gated ion channels (P2X) and heterotrimeric G protein-coupled receptors (P2Y). P2Y receptors have become recognised as the important regulators of carcinogenesis, endothelial regulation, and blood flow regulation (Buxton *et al*, 2001; Burnstock, 2006; White and Burnstock, 2006). Little is known about the role of P2Y receptors (P2YRs) in angiogenesis with only a handful of reports providing evidence supporting this notion. We have shown earlier that P2YR signaling promotes angiogenic properties such as endothelial cell tubulogenesis (Rumjahn *et al*, 2007), whereas others have reported P2YR-mediated migration (Satterwhite *et al*, 1999; Kaczmarek *et al*, 2005) and permeability (Tanaka *et al*, 2004, 2006). Activated P2Y_2_ receptors can transactivate vascular endothelial growth factor receptor-2 (VEGFR-2), suggesting a direct link between extracellular nucleotides and established tumour angiogenesis signaling (Seye *et al*, 2004). VEGFR-2 mediates the majority of the angiogenic and permeability-enhancing effects of VEGF (Shibuya, 2006). Given this evidence, we hypothesised that endothelial P2YR signaling interacts to regulate VEGFR-2 signaling. Here, we provide evidence that P2Y_1_R stimulation of human endothelial cells activates VEGFR-2 intracellular signaling to stimulate endothelial cell tubulogenesis, a direct *in vitro* measure of angiogenesis. These data suggest that tumour-mediated angiogenesis signaling may be, in part, mediated by nucleotide receptor activation of the VEGFR-2 pathway and may effectively lower the local requirement for VEGF.

## Materials and methods

### Cell culture

Human cardiac endothelial cells (HCECs) were earlier isolated by Fluorescence Activated Cell Sorting for CD31 (PECAM) and immortalised by human telomerase reverse transcriptase (hTRT). Cloned human cord blood endothelial colony forming cells (ECFCs) were purchased from Dynacell Life Sciences (Spring House, PA, USA) and used experimentally between passages 6 and 13. The HCEC population represents an immortalised cell line, whereas the ECFC population represents a primary cell line.

Human cardiac endothelial cells were grown in Dulbecco's Modified Eagle's Medium (HyClone, Logan, UT, USA) supplemented with 10% foetal bovine serum ((FBS) Atlanta Biological, Lawrenceville, GA, USA), penicillin–streptomycin (1500 Ul^−1^–100 mgl^−1^), and 0.5mgl^−1^ fungizone (Invitrogen, Carlsbad, CA, USA). Human ECFCs were grown in endothelial growth media-2 ((EGM-2) Clonetics, East Rutherford, NJ, USA) supplemented with 10% FBS (v/v), penicillin–streptomycin, and fungizone as above. EGM-2 without angiogenic growth factors (EBM-2, Clonetics) and with low serum (2% FBS) was used during angiogenic experimental treatments. Cells were grown and maintained at 37°C in a humidified atmosphere with 5% CO_2_/95% air.

### *In vitro* angiogenesis scoring technique

A representative endothelial cell tubulogenesis (angiogenesis) score for each condition was obtained by analysing digital images ( × 100) collected from the central pointing corners of quadrants I–IV in each culture well and averaging the four scores. As described earlier (Rumjahn *et al*, 2007), an angiogenesis score (*s*) represents the product of mean number of branch points (*bp*) multiplied by mean pixel length (*l*) multiplied by mean pixel cell surface area (*a*). Thus, *s=bp* × *l* × *a*.

### Effect of disrupting VEGF signaling in P2Y receptor-mediated angiogenesis

Human cardiac endothelial cells (3 × 10^4^) on collagen-coated plates were incubated for 24 h with P2Y receptor agonists ATP (P2Y_1/2_R; 100 *μ*M) and 2-methyl-thio-ATP (2MS-ATP) (P2Y_1_R; 10 *μ*M). EC tubulogenesis was also observed in the presence of 1 *μ*M SU1498 (∼IC_50_ of VEGFR-2 tyrosine kinase inhibitor (Strawn *et al*, 1996); Sigma, St Louis, MO, USA) with either 100 *μ*M ATP or 10 *μ*M 2MS-ATP. EGM-2 was employed as a positive control, whereas non-treatment controls were performed for normalisation and comparison. EGM-2 stimulated growth was also observed in the presence of 1 *μ*M SU1498 as a VEGFR-2 inhibition control. The use of 10 *μ*M 2MS-ATP or 100 *μ*M ATP in promoting EC tubulogenesis was maximal, consistent with known desensitisation of P2Y_1/2_ receptors at higher agonist concentrations (Ralevic and Burnstock, 1998; Rumjahn *et al*, 2007). SU1498 was chosen as it is at least 100-fold more selective for VEGFR-2 kinase compared with other receptor tyrosine kinases such as platelet-derived growth factor receptor, epidermal growth factor (EGF) receptor, and human epidermal growth factor receptor 2 (Strawn *et al*, 1996).

Endothelial colony forming cells (5 × 10^4^) plated on growth factor reduced (GFR) Matrigel (BD Biosciences, Bedford, MA, USA) were incubated for 24 h with varying concentrations (0.05–10 ng/ml; 1.31–262 pM) of VEGF-A (VEGF; Sigma). Non-treatment controls were performed for normalisation and comparison. The experimentally determined maximal VEGF stimulation of EC tubulogenesis (262 pM) was used as a positive control in all subsequent P2Y/VEGF experiments.

Endothelial colony forming cells (5 × 10^4^) grown on GFR Matrigel were incubated for 24 h in the presence of 10 *μ*M 2MS-ATP with or without 1 *μ*M SU1498. VEGF (262 pM) in EBM-2 was used as a positive control and was also used in the presence of 1 *μ*M SU1498 to show the VEGFR-2-specific promotion of EC tubulogenesis. Non-treatment controls were performed for normalisation and comparison.

### Effect of P2Y_1_ receptor activation on VEGFR-2 activation

Endothelial colony forming cells were first grown to ∼75% confleuncy on collagen-coated plates and then switched to EBM-2 supplemented with 0.5% FBS (v/v) overnight. ECFCs were next stimulated with either VEGF (0.0262 or 2.62 nM) or 2MS-ATP (10 nM or 10 *μ*M) for 10 min. The cells were collected in cold NP-40 lysis buffer. This cell lysate was centrifuged at 10 000 **g** to remove cellular debris.

Cell lysate supernatants (150 *μ*g) were incubated with KDR/VEGFR-2 specific antibody (0.3 *μ*g rabbit IgG; Chemicon, Rockford, IL, USA) and protein A/G agarose beads (20 *μ*l; Santa Cruz Biotechnology Inc., Santa Cruz, CA, USA). Proteins were separated by electrophoresis on 10% polyacrylamide gels (Bio-Rad, Hercules, CA, USA) and transferred to nitrocellulose membranes. The membranes were then labeled for either overall phosphotyrosines (1 : 1000 mouse IgG; Chemicon) or VEGFR-2 phosphotyrosine 1175 (1 : 500 rabbit IgG; Cell Signaling Technology, Danvers, MA, USA). Respective secondary antibodies conjugated to either *Alexa Fluor 680* or *Alexa Fluor 800* fluorescent dye (1 : 100 000; Invitrogen) were used for detection. Antibody incubations were carried out in 1 : 1 Odyssey blocking buffer (Licor Biosciences, Lincoln, NE, USA) and PBS with 0.1% Tween-20 (v/v). Bands were visualised using the Odyssey Infrared Imaging System (V2.04). VEGFR-2 levels were checked for equal loading.

### Effect of 2-Methyl-Thio-ATP signaling on angiogenesis

Endothelial colony forming cells (5 × 10^4^) on GFR Matrigel-coated plates were incubated for 24 h with various concentrations of 2MS-ATP (0.1–10 *μ*M in half-log increments). VEGF (262 pM) was used as a positive control, whereas non-treatment controls were performed for normalisation and comparison.

### Effect of 2-Methyl-Thio-ATP and vascular endothelial growth factor signaling on angiogenesis

Endothelial colony forming cells (5 × 10^4^) on GFR Matrigel-coated plates were incubated for 24 h with various concentrations of 2MS-ATP (0.1–10 *μ*M in half-log increments) in the presence of sub-maximal VEGF stimulation (ECFC tubulogenesis ∼EC_50_ as experimentally determined; 70 pM) in order to determine potential co-activation between these angiogenic molecules. VEGF (262 pM) was used as a positive control, whereas non-treatment controls provided normalisation and comparison.

### Statistical analyses

All graphs were prepared using Prism Graphing Software (V5.01; GraphPad Software, San Diego, CA, USA) and statistical analyses were carried out using InStat Statistical Software (V3.06; GraphPad Software), with *P*⩽0.05 considered significant. Significance was determined using non-parametric analysis of variance (Kruskal–Wallis) with Dunn's multiple comparisons post-test or non-parametric *t*-test (Mann–Whitney). Data points and error bars represent means±s.e.m. ^*^P⩽0.05; ^**^P⩽0.01; ^***^P⩽0.001 (*vs* negative control, unless otherwise indicated); ^#^*P*⩽0.05 (2MS-ATP alone *vs* 2MS-ATP+VEGF combination).

## Results

### Endothelial P2Y_1/2_ receptors mediate *in vitro* angiogenesis through VEGF signaling

**Figure 1:**
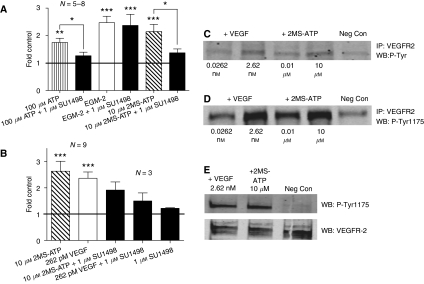
Endothelial P2Y receptor-mediated *in vitro* angiogenesis uses vascular endothelial growth factor (VEGF) signaling. Inhibition of VEGFR-2 intracellular signaling by SU1498 suppressed the pro-angiogenic potential of P2Y_1/2_ receptor agonists ATP and/or 2-methyl-thio-ATP (2MS-ATP) during a 24 h EC tubulogenesis assay. (**A**) Control mean=979.4±403.6 angiogenesis units. Negative control **A**; HCECs incubated in CDMEM supplemented with 2% FBS and 0.01% (v/v) DMSO. The angiogenic stimulation control used was endothelial growth media-2 (EGM-2). (**B**) Control mean=817.4±31.1 angiogenesis units. Negative control **B**; ECFCs incubated in EBM-2 supplemented with 2% FBS and 0.01% (v/v) DMSO. The angiogenic stimulation control used was EBM-2 containing VEGF, which was also suppressed by SU1498. (**C**–**E**); ECFCs treated with either VEGF (natural VEFGR-2 agonist) or 2MS-ATP (P2Y_1_R agonist) for 10 min observed respective phosphorylation and trans-phosphorylation of VEGFR-2. Samples were immunoprecipitated for VEGFR-2 and western blotted for (**C**) overall phosphotyrosines or (**D**) phosphotyrosine 1175. (**E**) Samples were directly western blotted for VEGFR-2 and phosphotyrosine 1175. Negative control **C** and **D**; ECFCs incubated in EBM-2 supplemented with 0.5% FBS. Negative control **E**; EBM-2 with 1% FBS. ^*^P⩽0.05; ^**^P⩽0.01; ^***^P⩽0.001.

**Figure 2:**
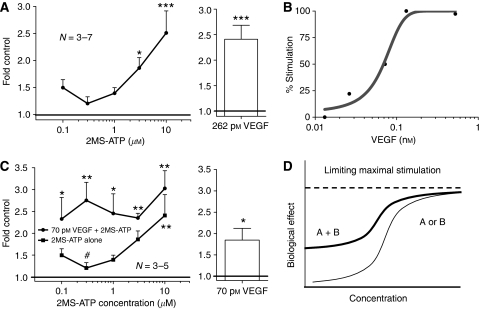
2-methyl-thio-ATP (2MS-ATP) and vascular endothelial growth factor (VEGF) cooperatively promote *in vitro* angiogenesis. P2Y_1_ or VEGF signaling alone, as well as together stimulated EC tubulogenesis over a 24 h duration. (**A**) ECFCs treated with varying amounts of 2MS-ATP produced a dose-dependent stimulation of tubulogenesis. Control mean=1292.8±65.1 angiogenesis units. Negative control **A**; ECFCs incubated in EBM-2 supplemented with 2% FBS. The angiogenic stimulation control used was EBM-2 containing VEGF. (**B**) VEGF (natural VEFGR-2 agonist) produced a dose-dependent stimulation of tubulogenesis. Angiogenic responses varied between 1.25- and 3.00-fold control (defined as 0 and 100% stimulation). Negative control **B**; ECFCs incubated in EBM-2 supplemented with 2% FBS. Curve trace was calculated using a non-linear fit of the data employing an equation describing a sigmoidal curve. (**C**) ECFCs incubated with varying concentrations of 2MS-ATP combined with a constant sub-maximal level of VEGF (apparent tubulogenesis EC_50_ of 70 pM) produced additive stimulation of EC tubulogenesis only at lower concentrations of 2MS-ATP. Control mean=655.7±81.8 angiogenesis units. Negative control **C**; ECFCs incubated in EBM-2 supplemented with 2% FBS. Fold control – 1.00 equals non-stimulated (negative) control. (**D**) Hypothetical curve illustrating two molecules promoting a biological response (e.g., angiogenesis) *via* convergent signaling pathways, which limits the potential of a larger response at higher concentrations. ^*^P⩽0.05; ^**^P⩽0.01; ^***^P⩽0.001.

As P2Y receptors have earlier been reported to interact with VEGFR-2 (Seye *et al*, 2004), we investigated whether nucleotides and VEGF-induced angiogenesis involved a common signaling pathway. HCECs incubated with P2Y_1/2_ receptor agonists (100 *μ*M ATP or 10 *μ*M 2MS-ATP) for 24 h showed a ∼2.0-fold angiogenic stimulation (*P*⩽0.01; Figure 1A) that was suppressed back to ∼1.3-fold control by 1 *μ*M SU1498. The ∼IC_50_ of SU1498, a VEGFR-2 tyrosine kinase inhibitor, was used to limit non-specific inhibition of other RTKs (e.g., platelet-derived growth factor receptor and epidermal growth factor receptor) as reported earlier with higher concentrations of this agent (Strawn *et al*, 1996). The EGM-2 control produced a ∼2.5-fold increase over control (*P*⩽0.001; Figure 2), which, interestingly, was not affected by the addition of 1 *μ*M SU1498. EGM-2 (a proprietary cocktail) includes a mix of various angiogenic factors, including VEGF.

Using primary human endothelial cells, we also observed that P2Y_1_ receptor activation stimulates VEGFR-2-mediated EC tubulogenesis. ECFCs incubated with 10 *μ*M 2MS-ATP (P2Y_1_ receptor agonist) for 24 h exhibited a ∼2.6-fold angiogenic stimulation (*P*⩽0.001; Figure 1B) that was suppressed back to ∼1.9-fold control by 1 *μ*M SU1498. The VEGF control (262 pM) observed a ∼2.4-fold increase over control, which was suppressed back to ∼1.5-fold control by 1 *μ*M SU1498. The SU1498 control showed minimal angiogenic effect alone.

### Activated endothelial P2Y_1_ receptors transphosphorylate VEGFR-2

It is well known that ligand binding of a RTK (e.g., VEGFR-2) induces dimerisation and subsequent activation of the receptor, resulting in the autophosphorylation of tyrosine residues in its cytoplasmic domain (Arora and Scholar, 2005). Knowing that P2Y_1_R-mediated EC tubulogenesis uses VEGFR-2 intracellular signaling, we further asked if P2Y_1_R activation by 2MS-ATP would transphosphorylate (i.e., transactivate) VEGFR-2. ECFCs stimulated with VEGF (0.0262 or 2.62 nM) showed phosphorylation of VEGFR-2 in a dose-dependent manner (Figure 1C; lanes 1 and 2), consistent with the known activation of this receptor by its natural ligand. ECFCs stimulated with 2MS-ATP (0.01 and 10 *μ*M) also exhibited a similar dose-dependent phosphorylation of VEGFR-2 (Figure 1C; lanes 3 and 4). The non-stimulated (negative) control showed a minimal level of basal VEGFR-2 tyrosine phosphorylation (Figure 1C; lane 5).

Phosphorylated tyrosine 1175 of VEGFR-2 is a binding site for the SH2 domain of phospholipase C, which is an important mediator of VEGFR-2-induced angiogenesis (Shibuya, 2006). We further observed that ECFCs stimulated with either VEGF or 2MS-ATP phosphorylated VEGFR-2 Tyr1175 in a similar dose-dependent fashion (Figure 1D and E). Human umbilical vein endothelial cells also showed similar levels of VEGFR-2 tyrosine phosphorylation by VEGF and 2MS-ATP (data not shown).

### Endothelial P2Y_1_ receptor activation stimulates EC tubulogenesis in a dose-dependent manner

We observed that 2MS-ATP promotes a dose-dependent angiogenic response with significant stimulation seen at higher concentrations of ⩾3 *μ*M 2MS-ATP, ∼2.5-fold increase over control (*P*⩽0.05; Figure 2A). We observed earlier that ⩾10 *μ*M 2MS-ATP does not provide additional angiogenic stimulation (Rumjahn *et al*, 2007), therefore an apparent tubulogenesis EC_50_ would be ∼3 *μ*M. The VEGF control (262 pM) stimulated angiogenesis similar to that seen with 10 *μ*M 2MS-ATP.

### VEGF stimulates *in vitro* angiogenesis in a dose-dependent manner

Endothelial colony forming cells incubated with varying concentrations of VEGF (0.00131–0.524 nM) over 24 h also exhibited a dose-dependent increase in EC tubulogenesis with maximal response at levels ⩾∼0.262 nM (Figure 2B). On the basis of these observations, an apparent VEGF EC_50_ of ∼70 pM was used as a sub-optimal level of VEGF stimulation of tubulogenesis.

### 2MS-ATP and VEGF use a common angiogenic pathway

To further investigate the notion of P2Y and VEGF cooperative signaling, we incubated ECFCs with varying concentrations of 2MS-ATP (0.1–10 *μ*M) in the presence of constant tubulogenesis EC_50_ concentration for VEGF (70 pM). We observed that the combination of sub-optimal levels of 2MS-ATP and VEGF do indeed promote a significant dose-dependent angiogenic response when compared with 2MS-ATP alone and control (*P*⩽0.05; Figure 2C). Lower concentrations of 2MS-ATP (<3 *μ*M) in combination with VEGF (70 pM) exhibited additive-like angiogenic stimulation, whereas higher concentrations (⩾3 *μ*M 2MS-ATP) produced a more saturated and less than additive promotion of EC tubulogenesis. VEGF at 70 pM provided ∼51% of maximal VEGF stimulated EC tubulogenesis (compared with 262 pM), consistent with our preliminary determination of the EC_50_.

## Discussion

We observed earlier that pathologically secreted NDPK stimulates angiogenesis in a nucleotide-dependent manner principally by P2Y_1_R (Rumjahn *et al*, 2007). Supporting this notion of P2Y-mediated angiogenesis, it has been reported that endothelial P2Y_2_R-mediated VEGFR-2 activation stimulates the expression of pro-inflammatory vascular cell adhesion molecule 1 (VCAM-1) (Seye *et al*, 2003, 2004). Inflammation and angiogenesis share common mechanisms and are often seen concurrently, especially in carcinogenesis. An appropriate example would be the use of VCAM-1 in the recruitment of monocytes (undifferentiated macrophages) and the differentiation of these cells into tumour-associated macrophages, which can provide a microenvironment conducive to tumour growth, metastasis, and of course angiogenesis, as especially prevalent in breast and prostate cancers (Pollard, 2004).

We suggest that P2YR-mediated VEGFR-2 activation can promote tumour angiogenesis indirectly, as well as stimulate direct angiogenic effects on the endothelial cells where the original P2Y/VEGF signaling occurs. We investigated the latter notion and observed the inhibition of ATP and 2MS-ATP-mediated EC tubulogenesis by VEGFR-2 tyrosine kinase inhibitor SU1498. This showed that VEGFR-2 intracellular signaling is a substantial component of P2YR-mediated *in vitro* angiogenesis. The unaffected angiogenic potential of EGM-2 (includes angiogenic factors in addition to VEGF) by SU1498 is consistent with the presence of multiple angiogenic pathways that can compensate for impaired VEGF signaling. As this P2Y-mediated VEGFR-2 signaling was observed in immortalised endothelial cells (HCECs), we further showed that this signaling also exists in primary endothelial cells (ECFCs). In the presence of a more relevant VEGF angiogenic control, SU1498 (∼IC_50_ tyrosine kinase activity) produced a ∼60% reduction in stimulated EC tubulogenesis indicating a tight association between VEGFR-2 tyrosine kinase activity and our detection of EC tubulogenesis. VEGF (0.262 nM) exhibited maximal induction, whereas higher concentrations (>1.31 nM VEGF) exhibited signs of desensitisation, consistent with known internalisation and degradation of activated VEGFR-2 (Ewan *et al*, 2006).

In addition to our functional angiogenesis data we observed that P2Y_1_R activation by 2MS-ATP induces tyrosine phosphorylation, general and tyrosine 1175-specific, on the cytoplasmic domain of VEGFR-2 similar to that seen by its natural ligand VEGF. Tyr1175 of VEGFR-2 and its phosphorylation have been shown to be the important regulators of VEGF-dependent angiogenic signaling (Holmqvist *et al*, 2004; Sakurai *et al*, 2005; Shibuya, 2006). This P2YR-mediated transphosphorylation and transactivation of VEGFR-2 provides further evidence that P2YR uses VEGF signaling to stimulate angiogenesis. This is the first report of P2Y_1_R activation of VEGFR-2 angiogenic signaling.

P2Y_1_R activation of VEGFR-2 signaling was further investigated by showing that varying levels of P2Y_1_R activation produces a dose-dependent angiogenic response. When sub-maximal VEGF signaling (experimentally determined EC_50_ of ∼70 pM) was subtracted from the combination of 2MS-ATP and VEGF, concentrations of 2MS-ATP ⩾3 *μ*M observed angiogenesis levels less than that seen from 2MS-ATP treatment alone (less than additive effect). This can be conceptually explained when considering the stimulation of a biological response (e.g., EC tubulogenesis) by two molecules using the same signaling pathway, as illustrated in Figure 2D. This dose-dependent saturation of VEGF angiogenic signaling by 2MS-ATP further suggests that P2Y_1_R-mediated angiogenesis is dependent on VEGFR-2 signaling and moreover, that VEGFR-2 does not need its natural ligand to become activated. P2Y receptors may additionally use their own separate signaling pathways independent of VEGFR-2 to promote angiogenesis, and it is possible that these independent pathways converge at one or more distal effectors.

**Figure 3:**
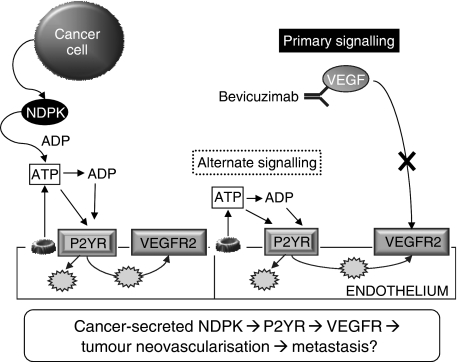
Putative role of extracellular nucleoside diphosphate kinase (NDPK) and P2Y receptor (P2YR)/vascular endothelial growth factor receptor (VEGFR-2) activation in angiogenesis. We have observed breast cancer-secreted NDPK-B to be a significant contributor in promoting angiogenesis. Extracellular NDPK would modulate nucleotides such as elevating ATP levels (Rumjahn *et al*, 2007). Our current data supports a scenario where P2Y purinergic receptor activation above an unknown threshold would produce conditions favourable to pathological angiogenesis. Moreover, this P2Y angiogenic signaling would cooperate with VEGF angiogenic signal. This posits the notion of dual inhibition of VEGF signaling through sequestering extracellular VEGF levels (i.e., Bevicuzimab) as well as blocking P2YR-dependent activation of VEGFR-2.

We now further expand our original hypothesis to include VEGFR-2 signaling as a significant contributor to P2YR-induced angiogenesis, which cancer cells can exploit to pathological advantage by secretion of NDPK (Figure 3). Our demonstration of productive interaction between P2Y and VEGF signaling predicts that low levels of ATP and VEGF can cooperate to stimulate considerable angiogenesis. Dynamic regulation of these two molecules (ATP>VEGF or VEGF>ATP) may be used to produce similar angiogenic responses in a temporal- and environment-dependent manner. With respect to pathological angiogenesis, tumour vasculature is more sensitive to VEGF withdrawal as it mimics immature vessels that are extremely dependent on VEGF signaling (Benjamin *et al*, 1999). Thus we propose that dual inhibition of P2YR and VEGFR-2 signaling may provide an effective mode of combinational anti-angiogenic therapy. It is interesting that, this P2YR activation of VEGFR-2 intracellular signaling may also, in part, explain constitutive VEGFR-2 activation in the absence of exogenous VEGF (Lee *et al*, 2007). Therefore, P2YR-VEGFR2 signaling may be important in describing and understanding VEGF signaling required for endothelial homoeostasis in both tumour as well as normal vasculature.
